# Non-invasive screening for subclinical liver graft injury in adults via donor-specific anti-HLA antibodies

**DOI:** 10.1038/s41598-020-70938-7

**Published:** 2020-08-28

**Authors:** Anne Höfer, Danny Jonigk, Björn Hartleben, Murielle Verboom, Michael Hallensleben, Michael P. Manns, Elmar Jaeckel, Richard Taubert

**Affiliations:** 1 Department of Gastroenterology, Hepatology and Endocrinology, Hannover Medical School, Hannover, Germany; 2 Integrated Research and Treatment Center Transplantation (IFB-Tx), Hannover Medical School, Hannover, Germany; 3 European Reference Network On Hepatological Diseases (ERN RARE-LIVER), Hannover, Germany; 4 Institute for Pathology, Hannover Medical School, Hannover, Germany; 5 Institute for Transfusion Medicine and Transplant Engineering, Hannover Medical School, Hannover, Germany

**Keywords:** Hepatology, Translational immunology

## Abstract

The majority of liver grafts exhibit abnormal histological findings late after transplantation, even when liver enzymes are normal. Such subclinical graft injuries were associated with rejection and fibrosis progression in recent studies. The identification of non-invasive biomarkers for subclinical graft injury might help to individualize immunosuppression. Therefore, graft injury was assessed in 133 liver biopsies with normal/near normal liver enzymes from a prospective liver biopsy program. Cytokeratin-18 cell death marker (M65) and donor specific anti-HLA antibodies (DSA) were measured as non-invasive markers in paired plasma samples in addition to routine parameters. M65 was associated with subclinical graft injury but this association was too weak for reasonable clinical application. DSA positivity was associated with more graft inflammation (OR = 5.4) and more fibrosis (OR = 4.2). Absence of DSA excluded fibrosis in 87–89%, while presence of DSA excluded histological criteria for immunosuppression minimization attempts in 92–97%. While CK18 cell death marker had no diagnostic value for the detection of subclinical liver graft injury, DSA testing can help to preselect patients for immunosuppression reduction in case of DSA negativity, while DSA positivity should prompt elastography or liver biopsy for the assessment of subclinical graft injury.

## Introduction

Long-term survival after liver transplantation (Ltx) did not improve for the last three decades. Approximately 25% of deaths beyond year one after liver transplantation were associated with the long-term intake of immunosuppression, e.g. malignancies and infections, while liver graft dysfunction was only responsible for 12% of deaths^1^. An individualization of immunosuppression promises a better balance of necessary control of alloreactivity and side effects of immunosuppressants. Approaches for such a personalized medicine are safer after Ltx than after other solid organ transplants, because of lower rates of chronic and antibody-mediated rejection and the highest rates of spontaneous operational tolerance.

However, it is still difficult to monitor the control of alloreactivity after liver transplantation, because liver enzymes are rather insensitive to detect subclinical graft injuries. Protocol liver biopsies, the current gold standard for the detection of subclinical graft injury, could fill this gap of sensitivity, but lack specificity, because subclinical histological abnormalities are found in the majority of livers allografts late after transplantation even in patients with a stable long term course^2–4^. The relevance of many subclinical abnormalities remained unclear, because typical features of T cell-mediated rejection (TCMR) were less frequent late after transplantation. On the other hand, patients that achieved spontaneous operational tolerance in clinical trial had not necessarily a complete absence of inflammation in screening biopsies^5,6^. Just recently, a first consensus document describing pattern of chronic antibody-mediated rejection (cAMR) after Ltx was published^7^.

Unfortunately, non-invasive markers predicting subclinical graft injury are still missing. A promising candidate is cytokeratin-18 (CK18) that is released during hepatocyte cell death. Serum levels of CK18 and its cleaved fragments can help to diagnose non-alcoholic steatohepatitis and have prognostic relevance e.g. during acute liver failure or primary biliary cholangitis^8–10^.

Donor specific anti-HLA antibodies (DSA) are well known to be associated with graft injury and rejection^4,11–21^. Although graft and patient survival of DSA positive liver transplanted patients is reduced, the association between DSA positivity and graft outcome is milder than after e.g. kidney transplantation^14–16,22,23^ and DSAs can even be found in operational tolerant patients^24,25^.

The aim of this study was to test the association of blood levels of CK18 and DSAs with defined patterns of subclinical graft injury with clinical relevance in an adult protocol liver graft biopsy program.

## Results

### Patients’ characteristics

**Figure 1:**
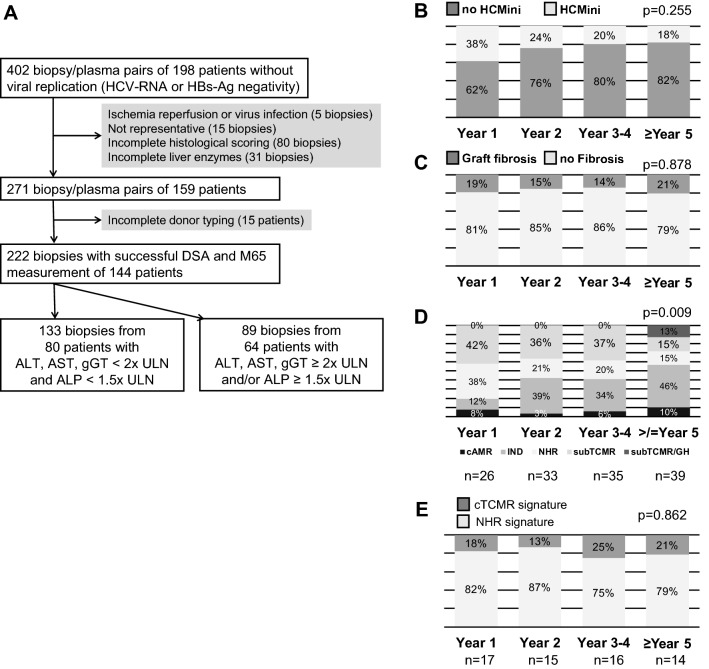
Biopsy selection and time course of subclinical histological findings. (**A**) Flow chart outlining availability and selection of biomaterial for this study. (**B**–**E**) Prevalence of histological findings (histological criteria for minimization of immunosuppression (HCMini) and at least moderate graft fibrosis), diagnosis (cAMR = possible chronic antibody-mediated rejection; IND = indeterminate findings; NHR = no histological rejection; subTCMR = subclinical T cell-mediated rejection; GH = graft hepatitis not fulfilling cAMR criteria) and graft gene expression signatures (cTCMR = clinical relevant T cell mediated rejection) in liver biopsies with normal/marginally elevated liver enzymes (n = 133) over time. P-values of Chi^2^ test of the prevalence of time are depicted.

Patients for this study were transplanted between 1990 and 2015 and liver biopsies were taken between July 2009 and November 2017. In total 402 complete pairs of cryo-conserved plasma samples and liver biopsies were collected. Sample selection for this retrospective analysis is shown in Fig. 1A.

The aim of this study was to analyze the predictive capacities of non-invasive blood markers for histological findings with relevance for the individual immunosuppression management. Recently, the first histological criteria conducive to minimization of immunosuppression (HCMini) were published in a Banff consensus document^7^. Therefore, similar thresholds for liver enzyme elevations were chosen (ALT and AST and gGT < 2 × ULN and ALP < 1.5 × ULN) as in an adult prospective immunosuppression weaning trial^5^. Altogether 133 liver biopsies were taken while liver enzymes were within these limits (Fig. 1A). The majority of these liver biopsies were taken from patients after a first liver transplantation (n = 116) and 17/133 (13%) biopsies were taken after a retransplantation. The majority of patients were transplanted as adults and only 4/80 (5%) were transplanted during childhood or adolescence. Their clinical data are summarized in Table 1.

**Table 1:** Clinical characteristics.

Patient number	80
Biopsy number	133
Age at biopsy [years]	50 (19–69)
Age at Ltx [years]	47 (2–68)
Including pediatric Ltx ≤ 18 years [number (%)]	4/80 (5%)
Time since Ltx [months]	25 (3–298)
Male gender	49/80 (62%)
**Indication for Ltx**	
Autoimmune liver diseases	36%
Acute liver failure	15%
Alcoholic cirrhosis	11%
Hepatocellular carcinoma	8%
Viral hepatitis	8%
Cryptogenic	8%
others	14%
Including retransplantation	12.5%
**Immunosuppression at biopsy**	
CNI	51% Tacrolimus
	46% Cyclosporine A
mTOR	2% Everolimus
	2% Sirolimus
Mycophenolate	75%
Prednisolone	85%
Mono/double/triple immunosuppression	2/33/65%
**Biochemistry**	
AST [times ULN]	0.74 (0.29–1.91)
ALT [times ULN]	0.47 (0.13–1.97)
ALP [times ULN]	0.65 (0.19–1.54)
gGT [times ULN]	0.53 (0.13–1.98)
Bilirubin [times ULN]	0.48 (0.14–2.53)

On one side subclinical graft injury that may need more intense immunosuppression like subclinical graft hepatitis or subclinical T cell-mediated (subTCMR) or antibody-mediated rejection (AMR) should be identified in these patients with normal or only marginally elevated liver enzymes. Furthermore, an at least moderate graft fibrosis was chosen as a second histological entity for the subsequent analyses.

### Subclinical graft injury

The results of the pathological analysis of these 133 liver biopsies with normal/marginally elevated liver enzymes are summarized in Table 2. The most prominent finding was portal inflammation in 84–87%, while lobular inflammation was found in about half of the biopsies (55%). Similarly, fibrosis was mostly found in the portal (53%) and periportal area (50%) and much less frequent in the sinusoidal (17%) and perivenular compartment (18%). Both fibrosis scores (Ishak and LAF score) were stringently correlated with each other (Spearman rank correlation coefficient: 0.844, p < 0.001) as published recently^26^. Other abnormalities related to rejection like portal microvasculitis or central perivenulitis were only found in a minority of biopsies (15–18%). Similarly, relevant steatosis or steatohepatitis were only occasionally present. As expected, the graft injury and fibrosis stages in biopsies with normal/marginally elevated liver enzymes (n = 133) were significantly lower (Mann–Whitney U-test) than in those with higher liver enzyme elevation (n = 86) (data not shown). Only portal microvasculitis and steatosis/steatohepatitis were not significantly different.

**Table 2:**
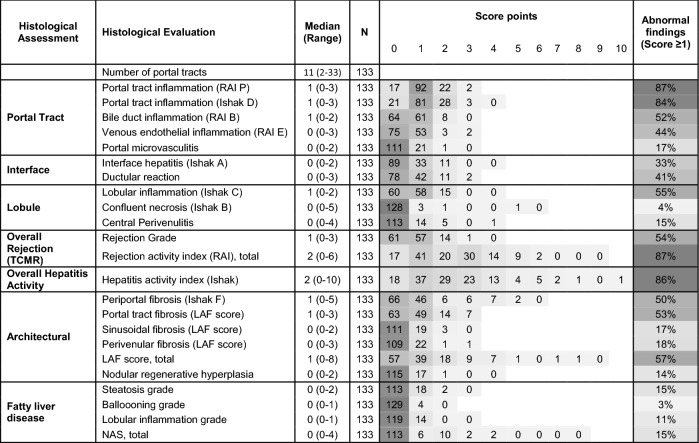
Histological characteristics of 133 liver biopsies with normal/marginally elevated liver enzymes.

SR = Spearman rank correlation coefficient; */** significant correlation on significance level of 0.05/0.01; grey scale increases with frequencies of score points.

Histological diagnoses in descending order were indeterminate findings (46/133; 35%), subTCMR (RAI ≥ 1 + 1 + 1; 42/133; 32%), no histological rejection (NHR: RAI ≤ 1 + 0 + 0 and Ishak ≤ 1 and without additional abnormalities; 30/133; 23%), possible cAMR (9/133; 7%), subTCMR with additional hepatitis features but not suggestive for cAMR (5/133; 4%) and PSC recurrence (1/133; 0.8%). Of these 133 biopsies, 32 (24% of all) were compatible with criteria of HCMini and 23 (17% of all) exhibited at least moderate fibrosis. HCMini and at least moderate graft fibrosis had stable frequencies over time (Fig. 1B + C). In contrast, histological diagnosis showed significant differences over time with an increase of indeterminate findings and graft hepatitis, while NHR declined later after transplantation (Fig. 1D).

Finally, graft gene expression from our recent study was available in 62/133 (47%) liver biopsies with normal/near normal liver enzymes of the current study. Based on the graft gene expression of 93 transcripts being associated with rejection and immune regulation after transplantation (Supplementary Table S1) we could recently identify two gene expression clusters in non-supervised cluster analysis. One cluster was enriched with biopsies showing clinical relevant TCMR (cTCMR) with liver enzyme elevation above two times upper limit of normal, while the other one was enriched with biopsies with no signs of rejection (no histological rejection = NHR)^19^. The gene expression pattern of twelve of the 133 biopsies (19%) of the current study fell into the cTCMR cluster, while the molecular signature of 50 biopsies (81%) fell into the NHR cluster. Although histological abnormalities increased over time, as indicated by the decrease of NHR (Fig. 1D), the molecular signatures of cTCMR and NHR are stable over time (Fig. 1E).

### Evaluation of liver enzymes and cytokeratin-18 as predictors of subclinical graft injury

**Figure 2:**
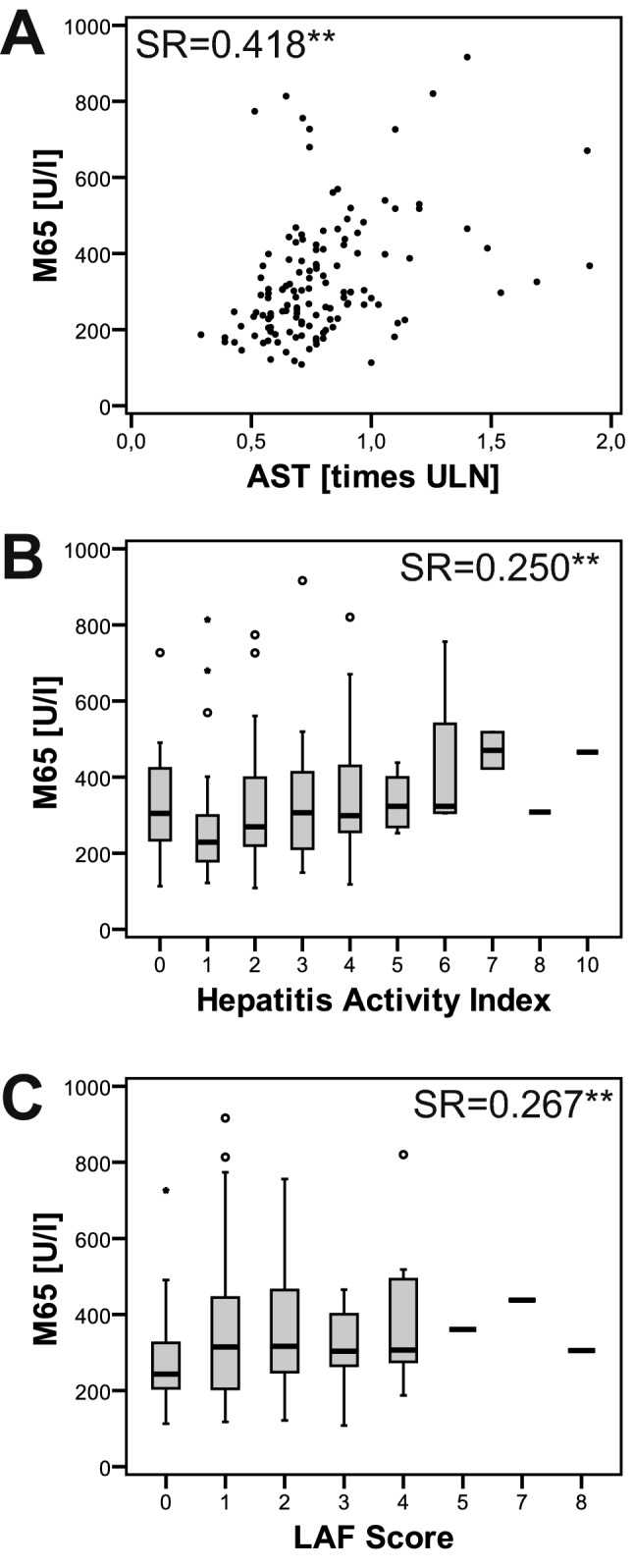
Association of cytokeratin-18 cell death biomarker with markers of subclinical liver graft. Cytokeratin-18 cell death marker (M65) was more stringently correlated with levels of liver enzymes, as exemplarily depicted for AST (A), than with histological scores for liver graft injury, as exemplified by the hepatitis activity index, or graft fibrosis, as assessed by the liver allograft fibrosis (LAF) score in 133 blood samples with normal/marginally elevated liver enzymes (B + C) (SR = Spearman rank correlation coefficient; **significant correlation at the 0.01 significance level).

M65 concentrations correlated closer with liver enzyme levels than with scores for histological graft injury such as hepatitis activity index and LAF score (Fig. 2A–C) in the 133 blood samples of patients with normal/marginally elevated liver enzymes.

In order to test if the blood parameters could predict the histological or transcriptional entities, AUROC analyses (HCMini, fibrosis, transcriptional signature of cTCMR) of the blood parameters were performed. Although, there were some significant associations of M65 or some liver enzymes, the associations were overall too weak for further reliable clinical applications or with cut-off values within the normal range (Supplementary Table S1). Of the routine liver function tests just ALT levels were relevantly associated with graft gene expression signature of cTCMR. However, the optimal cut-off was right in the normal range (0.443 times upper limit of normal) with a sensitivity and negative predictive value of 100%, a specificity of only 60% and a positive predictive value of 39%.

### Evaluation of DSA as non-invasive predictors of subclinical inflammation

**Figure 3:**
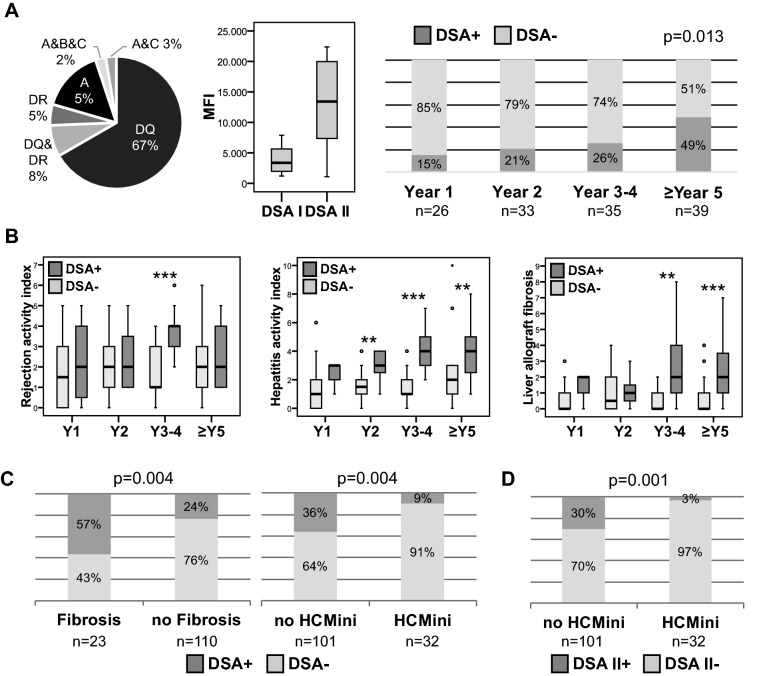
Association of DSA with subclinical graft injury and fibrosis. (**A**) DSA were detected in 39 of 133 samples with normal/marginally elevated liver enzymes. Specificity of DSA and MFI of class I and II DSA (left and middle panel). DSA frequency was increasing over time (right panel). (**B**) DSAs were associated with a progressive graft hepatitis and fibrosis over time (Y = year; **p < 0.01; ***p < 0.001). (**C**) Presence of DSA was associated with the severity of graft injury (HCMini = histological criteria of immunosuppression minimization) and at least moderate graft fibrosis. (**D**) Association of class II DSA with severity of graft injury. Further information on histological scores and DSA are listed in Table 3.

DSA were present in 39/133 (29%) blood samples with normal/near normal liver enzymes and in 20/80 (25%) patients. Class I DSA were found in 8/39 (21%) DSA positive samples from 4/20 (20%) DSA positive patients, while class II DSA were found in 31/39 (80%) DSA positive samples from 16/20 (80%) DSA positive patients. Specificities and MFI of DSA are outlined in Fig. 3A. While MFI of all DSA were above the threshold of 1,000, as recommended by a current position paper^27^, 27/39 (69%) DSA had MFI above 5,000.

The onsets of DSA (preexisting before transplantation or de novo after transplantation) could not be determined in our study, because pre-transplant samples were not available. However, longitudinal samples after transplantation in our biorepository could help to describe the persistence of DSA after transplantation. In this sense, 8/20 (40%) patients had DSA at all available time points. 4/20 (20%) patients were tested DSA positive at the only available time point after transplantation. 6/20 (30%) patients were tested DSA positive after being DSA negative at an earlier time point after transplantation. 2/20 (10%) patients were tested DSA positive and were DSA negative at a later time point. DSA frequencies significantly increased with time after transplantation (p = 0.013; Chi^2^ test; Fig. 3A).

DSA were found more often in male patients, later after transplantation, in patients with an autoimmune liver disease, in patients receiving cyclosporine A (CsA) rather than tacrolimus and in parallel to liver biopsies with more graft injury and more graft fibrosis (Table 3). The finding of more graft injury and more graft fibrosis in DSA positive biopsies persisted, when samples were matched for time after transplantation and gender as far as possible (see Supplementary Fig. S1 online). DSA positive patients had a higher hepatitis activity index and more graft fibrosis (Fig. 3B). Thereby, class I (n = 8) and class II (n = 31) DSA positive liver biopsies were not significantly different in terms of graft fibrosis/LAF score (p = 0.597), rejection activity index (p = 0.222) and hepatitis activity index (p = 0.162). A further assessment of an antibody-mediated graft injury by C4d staining was not possible, because > 98% of DSA positive samples had no traceable C4d deposition in the liver^19^.

**Table 3:** Clinical and histological characteristics of patients and liver biopsies according to DSA status.

	DSA+	DSA−	p
**Number of biopsies**	39	94	
Age at biopsy [years]	49 (22–67)	50 (19–69)	0.935
Age at Ltx [years]	45 (2–64)	48 (17–68)	0.571
Time since Ltx [months]	47 (5–298)	23 (3–85)	0.002
Gender [male %]	77	56	0.031*
**Immunosuppression at biopsy**			
CNI (Tac/CsA [%])	31/67%	60/38%	0.004
MTOR (Everolimus/Sirolimus [%])	0/5%	3/1%	0.400
Mycophenolate [%]	90%	85%	0.585
Prednisolone [%]	74%	74%	1.000
Mono/double/triple immunosuppression [%]	0/33/67%	2/34/64%	1.000
**Indication for Ltx**			
Autoimmune liver diseases	56%	28%	0.003
Acute liver failure	5%	19%	0.060
Alcoholic cirrhosis	5%	13%	0.232
Hepatocellular carcinoma	5%	11%	0.508
Viral hepatitis	13%	6%	0.298
Cryptogenic	5%	6%	1.000
others	10%	17%	0.428
**Biochemistry**			
M65 [U/L]	306 (118–820)	266 (108–916)	0.220
AST [times ULN]	0.74 (0.46–1.91)	0.74 (0.29–1.69)^§^	0.367
ALT [times ULN]	0.49 (0.27–1.70)	0.44 (0.13–1.97)^§^	0.159
ALP [times ULN]	0.88 (0.48–1.50)	0.60 (0.19–1.50)	< 0.001
gGT [times ULN]	0.58 (0.24–1.98)	0.50 (0.13–1.82)	0.506
Bilirubin [times ULN]	0.52 (0.24–1.62)^#^	0.47 (0.14–2.53)^§§^	0.220
**Histological scores**			
Rejection activity index	3 (0–6)	2 (0–6)	0.003
Hepatitis activity index	3 (1–8)	1 (0–10)	< 0.001
Interface hepatitis (Ishak A)	1 (0–2)	0 (0–2)	< 0.001
Confluent necrosis (Ishak B)	0 (0–2)	0 (0–5)	0.013
Lobular inflammation (Ishak C)	1 (0–2)	0 (0–2)	< 0.001
Portal tract inflammation (Ishak D)	2 (0–3)	1 (0–3)	< 0.001
Central Perivenulitis	0 (0–2)	0 (0–4)	0.006
Portal microvasculitis	0 (0–1)	0 (0–2)	0.001
**Fibrosis [score]**			
Periportal fibrosis (Ishak F)	1 (0–5)	0 (0–5)	< 0.001
Portal tract fibrosis (LAF score)	1 (0–3)	0 (0–3)	< 0.001
Sinusoidal fibrosis (LAF score)	0 (0–2)	0 (0–1)	< 0.001
Perivenular fibrosis (LAF score)	0 (0–3)	0 (0–1)	0.041
LAF score, total	2 (0–8)	0 (0–4)	< 0.001

*Fisher exact test; # n = 37; §n = 93; §§ n = 92.

MFI of DSA were positively correlated with hepatitis activity index (SR = 0.431; p < 0.01), thereby mostly with lobular inflammation (SR = 0.428; p < 0.01), graft fibrosis/LAF score (SR = 0.376; p < 0.05), thereby especially with sinusoidal fibrosis (SR = 0.476; p < 0.01), portal microvasculitis (SR = 0.346; p < 0.05), ductular reaction (SR = 0.328; p < 0.01) and nodular regenerative hyperplasia (SR = 0.345; p < 0.01). Nonetheless, there was no significant difference of subclinical graft injury between samples with high (> 5,000) and low (< 5,000) MFI.

In terms of non-invasive prediction, HCMini was significantly associated with the absence of DSA (any DSA MFI > 1,000) (odds ratio (OR) = 5.4 with 95% confidence interval (CI): 1.5–18.9), while DSA positivity (any DSA MFI > 1,000) was significantly associated with graft fibrosis (OR = 4.2; CI: 1.7–10.7) (Fig. 3C). An association of DSA with the transcriptional signature of cTCMR was recently published^19^. DSA positivity (any DSA MFI > 1,000) mostly excluded HCMini in 92%, while DSA (any DSA MFI > 1,000) negativity excluded relevant graft fibrosis in 89%. The non-invasive diagnostic fidelity of DSA for graft fibrosis was not relevantly different, when only DSA with higher MFI (> 5,000) and/or DSA class II were considered (Supplementary Table S1). However, HCmini was excluded in up to 97% of liver biopsies, when only DSA class II were positive (Fig. 3D). A separate analysis for class I DSA was not performed, because there were only 8 samples with class I DSA.

## Discussion

While this was the first analysis of the predictive value of CK18 levels for graft injury after liver transplantation beyond hepatitis C^28^, the association of DSA with graft injury in general is a well described phenomenon^4,12,14,16,18,20,21^. However, we are not aware of another study with a specific focus on non-invasive capacities of both parameters in comparison to the routine blood markers to detect subclinical graft injury in adults. This is of clinical importance for an individualization of immunosuppressive regimens, because the presence of more advanced subclinical graft injury firstly limits attempts to lower immunosuppression^7^ and secondly might indicate insufficiently controlled alloreactivity with a “smoldering” graft damage^3,4^.

The threshold of approximately two times ULN instead of the normal range to distinguish between clinical and subclinical graft injury seems rather arbitrary. However, liver enzyme elevations above this threshold were independent risk factors for a reduced patient and graft survival in the setting of early subTCMR^29^. SubTCMR defined by liver enzymes below two times ULN had no progression to cTCMR or subsequent progressive fibrosis when left untreated later after transplantation^19,30^. The same thresholds were also used for the description of subclinical graft injury in other studies in adults^3^. Furthermore, the thresholds applied here were adapted from a prospective immunosuppression weaning trial after adult liver transplantation^5^ that were used similarly in an ongoing multicenter validation trials as well (LIFT trial, NCT02498977).

The patterns of subclinical graft injury in our transplantation center resembled what is published from other centers^3,4,21^. The most prominent findings were portal inflammation and fibrosis. Subclinical graft injury and fibrosis progressed over time after transplantation. Thereby, biopsies without evidence of rejection declined, while indeterminate findings increased over time. In contrast, subTCMR and possible cAMR had rather stable prevalence. This single center study also confirmed the common notion that graft injury has more hepatitis features later after transplantation^2,7^. The longitudinal sequence of inflammation, fibrosis and the association of graft injury with DSAs was also described in a recent pediatric study by Varma et al.^21^. Furthermore, biopsies in the present study were selected mostly regarding the level of liver enzymes. Thereby, biopsies with liver enzymes above 1.5–twofold ULN were excluded.

The progression of hepatitis features with time was accompanied by an increasing appearance of DSA. An increase of DSAs over time has been reported by many other but not all studies^11,12,21,31,32^. This association cannot help to unravel, whether DSA are cause or consequence of the graft injury. However, it is compatible with the two hit hypothesis, which proposes that inflammation in the graft induces upregulation of class II HLA molecules and this promotes a sensitization towards mismatched allo-HLA molecules^33^. In line with this hypothesis and in line with other studies, DSA were predominantly targeted against class II HLA in the current study. However, C4d deposition suggestive for AMR could not be found in any DSA positive liver biopsy.

The clinical relevance of DSA after liver transplantation is controversially discussed, because graft and patient survival is much less reduced in DSA positive liver recipients than after kidney transplantation^15,22,34^. Nonetheless, we and other groups found an association of DSA with graft injury^4,12,14,16,18–21^. Interestingly, the present data suggest that the association of DSA with subclinical graft injury is more evident later after transplantation, because mostly DSA positive recipients showed a progressive graft injury over time while DSA negatives did not. This has been reported in other studies without a special focus on subclinical findings as well^21,35^. In addition, higher MFI were associated with more subclinical graft injury e.g. portal microvasculitis and graft fibrosis, features being associated with antibody-mediated rejection^7^. Mostly DSA with high MFI, e.g. above 5,000–10,000, are associated with reduced graft and patient survival^13–15,22,23,36^. The majority of the DSA (69%) found in this cohort of patients with normal/near normal liver enzymes had DSA with high MFI (> 5,000), too.

The focus of this study was to evaluate if these associations of DSA with graft injury in patients with normal/near normal liver enzymes might support clinical decisions. Thereby, different MFI thresholds and different DSA classes were included separately, because an international position paper^27^ recommend MFI thresholds of 1,000–1,500 to define DSA positivity but relevant associations of DSA with reduced graft or patient survival after liver transplantation were mostly found for DSA with higher MFI as mentioned above. The absence of DSA in the present patient cohort had an OR of 5.4 for the presence of HCMini according to the Banff guidelines^7^ justifying attempts to further reduce immunosuppressants. The diagnostic fidelity of DSA positivity for the exclusion of HCMini could be further increased, when only class II DSA irrespective of high or low MFI were considered. A limitation of the present study in this aspect was the high prevalence of primary autoimmune liver diseases (36%, Table 1), that were usually excluded from immunosuppression withdrawal studies^5,6^. Nonetheless, we think, it is appropriate to include patients with autoimmune background in such a mere association study and with the aim of an immunosuppression minimization and not a full withdrawal. However, autoimmune background was associated with more DSA before and after liver transplantation^35,36^.

On the other hand, the presence of DSA had an OR of 4.2 for the presence of at least moderate subclinical graft fibrosis. Thereby, we implemented the liver allograft fibrosis score, which was more in line with morphometric assessment of fibrosis than the Ishak or Metavir system^26^. In contrast to association of DSA and HCMini, the diagnostic fidelity of DSA to predict graft fibrosis could not be further increased when only class II DSA and/or DSA with high MFI were considered.

A limitation of the current study was the lack of information on pre-transplantation status of DSA, PRA and crossmatch. These pre-transplantation data are highly informative for the judgement of DSA regarding the appearance of humoral/antibody-mediated rejection. Unfortunately, it remains elusive, if the non-invasive diagnostic capacities of DSAs for subclinical inflammation and fibrosis in this study could be improved by focusing on de novo DSA. This aspect has to be addressed in subsequent studies.

Multiple studies analyzed potential non-invasive markers for the assessment of graft injury, mostly in the context of acute rejection^37^. Unfortunately, none of them reached a stage of broad clinical applicability. M65 was associated with subclinical graft injury, but the sensitivity of M65 in the liver transplantation setting was not relevantly higher than those of other liver enzymes. Graft injury after liver transplantation is more complex than a damage of hepatocytes, where cytokeratin-18 is mostly released from. Although M65 is associated with fibrosis in NAFLD before transplantation^8,38^, this association was rather weak after transplantation. The relevance of biliary graft injury beside hepatocellular graft injury is underlined by our finding, that ALP was the routine liver enzyme with the strongest association to graft injury and with the presence of DSA. Likewise, presence of DSA was associated with more bile duct changes (ductular reaction). Of note, this association was not strong enough to be used for a reasonable clinical non-invasive prediction. Multiple studies found associations of transient elastography with liver fibrosis stage in liver biopsies and transient elastography seems to be most appropriate to assess fibrosis non-invasively as compared to AST-to-platelet ratio index and fibrosis score 4^39^.

In summary, subclinical graft injury was a common finding on the histological and transcriptional level in protocol liver graft biopsies in adults. Cytokeratin-18 cell death marker (M65) were not suitable for non-invasive monitoring of subclinical graft injury. While DSA were significantly associated with subclinical graft injury, their low overall prevalence (29%) diminished their predictive capacity. However, the presence of DSA or class II DSA could help to exclude patients for a reduction of immunosuppression without the necessity of a liver biopsy. DSA positivity even in patients with normal/near normal liver enzymes should prompt an at least non-invasive assessment of fibrosis e.g. via elastography. However, in the end liver biopsies are still inevitable to reliably confirm subclinical graft injury for individualized immunosuppression guidance after adult liver transplantation.

## Material and methods

### Subjects

We included all adult liver recipients without a replicative viral hepatitis (HCV-RNA or HBs-Ag negativity) who underwent at least one liver biopsy and agreed to participate in our prospective liver allograft biorepository since 2008 (age at time of liver biopsy ≥ 18 years) as described recently^19^. Liver biopsies came from our protocol biopsiy program (intended time points: months 3 + 6 + 12 and then annually), or from patients with a liver biopsy because of elevated liver enzymes. A participation in the protocol biopsy program was voluntary and offered to all liver transplanted patients without contraindications, e.g. dilated bile duct, thrombocytopenia etc., even when they were transplanted before 2008. Likewise, all patients with a liver biopsy because of elevated liver enzymes were asked for a participation in the biorepository.

The study was approved by the Ethics Committee of Hannover Medical School, Hannover/Germany (protocol number 933 for project Z2 of comprehensive research center 738). Written informed consent was obtained from all subjects. All experiments were performed in accordance with relevant guidelines and regulations.

No organs or tissues were procured from prisoners. All liver transplantations of patients in this study were performed in Germany, where organ procurement and allocation are organized by Eurotransplant and the German Organ Transplantation Foundation.

### Liver biopsy specimens

Liver biopsies were performed percutaneously under local anesthesia. An approximately 5 mm portion of the needle biopsy was immediately preserved in Allprotect Tissue Reagent (QIAGEN) and then stored at − 80° C. The remaining cylinder was fixed in 4% neutral buffered formalin and embedded in paraffin wax.

### Histological grading and staging

Histological grading and staging was performed as described recently^19^. Sections of 2 µm thickness from liver allograft biopsies with a 17-gauge needle were stained with hematoxylin and eosin, elastic van gieson stain, periodic acid–Schiff stain, silver stain, Berlin blue stain and rhodanine stain. Histological examination and scoring for the rejection activity index (RAI)^7^, inflammation grade and fibrosis stage (Ishak score)^40^, central perivenulitis, portal microvasculitis, ductular reaction^7^, fatty liver disease^41^ and liver allograft fibrosis (LAF) score^26^ was performed by experienced liver pathologists in a blinded fashion.

The histological criteria conducive to minimization of immunosuppression (HCMini) were defined according to the last Banff consensus document^7^ as: portal tract inflammation ≤ 1, interface hepatitis ≤ 1, central perivenulitis ≤ 1, lobular/biliary inflammation/endothelialitis = 0, portal microvasculitis = 0, periportal fibrosis ≤ 3, any LAF score ≤ 1. At least moderate fibrosis was defined as: periportal fibrosis (Ishak F) ≥ 2 and/or any LAF score component ≥ 2.

### Detection of donor-specific anti-human leukocyte antigens antibodies

Blood samples were preferentially taken at the day of admission before the performance of the liver biopsy or within the first 24 h after the liver puncture. Blood plasma was cryo-conserved at – 80 °C.

DSAs were detected as described recently^19^. The recipient plasmas were screened for the presence of HLA class I/II antibodies using mixed HLA antigen-charged polysterene beads (LIFECODES LifeScreen Deluxe-LMX test Gen-Probe-Immucor, Stanford, CT, USA), using a multichannel flow array (Luminex, Austin, TX). A specification of HLA antibodies in sera with a positive screening result was performed using class I/class II single-antigen beads (LIFECODES Single Antigen-LSA test Gen-Probe-Immucor, Stanford, CT, USA). The tests were performed according to the manufacturer's instructions. The incidence of DSA positivity was analyzed using mean fluorescence intensity (MFI) threshold of 1,000 or more for the plasma antibodies against HLA and a positive match with donor HLA typing as recommended recently^27^. In case of multiple DSA, the highest MFI of all DSA is reported, but not the sum or the mean of MFI of all DSA. DSA, panel reactive antibodies (PRA) or crossmatch before transplantation are not part of the clinical work-up before liver transplantation at our center and were not available.

### Serological detection of caspase-cleaved and total cytokeratin-18

Caspase-generated neoepitopes of cytokeratin-18 (CK18) were measured in cryo-conserved plasma as recently published^8^. The M65 ELISAs were used according to the manufacturer’s instructions (Peviva, Bromma, Sweden).

The other laboratory parameters (aminotransferases and IgG) were measured in the course of the routine clinical surveillance of the patients.

### Liver tissue RNA extraction and processing

RNA was isolated from cryo-conserved liver biopsy fragments, reverse transcribed into cDNA and subsequent gene expression analysis was performed as described recently^19,42^.

### Statistics

Statistical analysis was performed with SPSS version 25 and GraphPad Prism 5.01 software as described recently^19^. The Mann–Whitney U test was used to compare quantitative data between two independent groups and the Kruskal–Wallis test with Dunn’s multiple comparison post hoc test for more than two groups. Fisher’s exact test, if possible, was used to compare contingency tables with two groups and the Chi square test was used to compare more than two groups. Spearman’s rank correlation coefficient (Spearman’s rho = SR) was used for correlation analyses.

P-values below 0.05 (two-tailed) were considered statistically significant in all analyses.

Further material and methods are listed in the supplementary information.

## Supplementary information

Supplementary file1.

## Data Availability

The data that support the plots within this paper and other findings of this study are available from the corresponding author upon reasonable request.

## Abbreviations

ALP: Alkaline phosphatase
ALT: Alanine transaminase
AMR: Antibody-mediated rejection
AST: Aspartate transaminase
cAMR: Possible chronic antibody-mediated rejection
CK18: Cytokeratin-18
CI: 95% Confidence interval
CNI: Calcineurin inhibitor
CsA: Cyclosporine A
cTCMR: Clinical T cell-mediated rejection
DSA: Donor-specific *anti*-human leukocyte antigen antibodies
gGT: Gamma-glutamyltransferase
GH: Graft hepatitis
HCMini: Histological criteria for minimization of immunosuppression
HLA: *Anti*-human leukocyte antigens
IND: Indeterminate histological findings
LAF: Liver allograft fibrosis
Ltx: Liver transplantation
MFI: Mean fluorescence intensity
NAS: Non-alcoholic fatty liver disease activity score
NHR: No histological rejection
OR: Odds ratio
PRA: Panel reactive antibody
PSC: Primary sclerosing cholangitis
RAI: Rejection activity index
subTCMR: Subclinical T cell-mediated rejection
Tac: Tacrolimus
TCMR: T cell-mediated rejection
ULN: Upper limit of normal
