# Supplementary material for: Non-invasive screening for subclinical liver graft injury in adults via donor-specific anti-HLA antibodies
Source: Sci Rep. 2020 Aug 28;10:14242. doi: 10.1038/s41598-020-70938-7 (PMC7455737; doi:10.1038/s41598-020-70938-7)

Non-invasive screening for subclinical liver graft injury in adults via donor-specific anti-HLA antibodies.

Anne Höfer[1, 2, 3], Danny Jonigk[4], Björn Hartleben[4], Murielle Verboom[5], Michael Hallensleben[5], Michael P. Manns[1, 3], Elmar Jaeckel[1, 2, 3], *Richard Taubert[1, 2, 3]

[1] Department of Gastroenterology, Hepatology and Endocrinology, Hannover Medical School, Germany

[2] Integrated Research and Treatment Center Transplantation (IFB-Tx), Hannover Medical School, Hannover, Germany

[3] European Reference Network on Hepatological Diseases (ERN RARE-LIVER)

[4] Institute for Pathology, Hannover Medical School, Germany

[5] Institute for Transfusion Medicine and Transplant Engineering, Hannover Medical School, Germany

*** Corresponding author email:**

PD Dr. Richard Taubert

taubert.richard@mh-hannover.de

Supplementary Information

Supplementary material and methods

Liver tissue RNA extraction and processing

Total RNA was extracted according to AllPrep DNA/RNA/Protein Mini protocol (Qiagen, Hilden, Germany) as descripted previously[1]. The samples of liver tissue homogenized in two steps, first in bead-milling disruption using Tissue Lyser and second using QIAshredder spin column. For isolation intact DNA, RNA and proteins the homogenization was preparing in buffer RLT, which inactivates DNases and RNases as well as proteases. The quality and quantity were assessed with the Agilent 2100 Bioanalyzer (Agilent Technologies, Santa Clara, CA, USA) and Nanodrop ND-1000, respectively. The RNA was then reverse transcribed into cDNA using the Transcriptor High Fidelity cDNA Synthesis Kit (Roche, Basel, Swizerland). Gene lists were taken from previous publications and the published results of the Molecular Microscope System from the Alberta Transplant Applied Genomic Centre (http://atagc.med.ualberta.ca/Research/GeneLists/Pages/default.aspx)[2-6] and supplemented with further genes associated with immune regulation (Supplementary Table S1).

A pre-amplification of cDNA over 14 cylces was performed using pooled TaqMan Assays and the TaqMan PreAmp Master Mix following manufacturer's protocol. RT-PCR was performed using the 48.48 and 96.96 Dynamic Array following manufacturer's protocol using a BioMark (Fluidigm Corporation, CA, USA). To quantify transcript levels, target gene Ct values were normalized using Ct values of GAPDH, GUSB, POLR2A as reference genes to generate -ΔCt values.

Analysis of intrahepatic gene expression data

Principal component analysis (PCA) and heat maps of -ΔCt values were performed using Qlucore Omics Explorer v3.3 (Qlucore, Lund, Sweden) as recently published[1]. For analysis p values were set of ≤0.049 to compare groups to each other for two group comparisons (t-test) and multi group comparison (F-test) (ANOVA). False discovery rate (FDR) calculated for correction of multiple t testing <0.1 was considered significant for all Qlucore analysis.

Supplementary Figures

Supplementary Figure S1: Association of DSA with subclinical graft injury and fibrosis after matching for time after transplantation.

Presence of DSA was associated with the severity of graft injury (HCMini) and at least moderate graft fibrosis in 95 samples with normal/marginally elevated liver enzymes after matching for time after transplantation of samples with and without DSA.

Supplementary Tables

Supplementary Table S1: Gene sets for the intrahepatic gene expression analysis.

**Rejection markers (RM)**[2]		**Endothelial cells markers (ECM)**[2]		**Immunoregulation and iron homeostasis markers (IM)**		**T cell exhaustion markers (TCEM) according to McKinney *et al.***[4]		**Spontaneous operational tolerance markers (SOTM) according to Bohne *et al.***[3]	
**Gene symbol**	**Official name**	**Gene symbol**	**Official name**	**Gene symbol**	**Official name**	**Gene symbol**	**Official name**	**Gene symbol**	**Official name**
ABCB1	ATP binding cassette subfamily B member 1	ADCY4	adenylate cyclase 4	BATF	basic leucine zipper ATF-like transcription factor	BCL2	BCL2, apoptosis regulator	CDHR2	cadherin related family member 2
ANXA2	annexin A2	CDH5	cadherin 5, type 2	CD3E	CD3e molecule, epsilon	BTLA	B and T lymphocyte associated	MIF	macrophage migration inhibitory factor
CCL19	C-C motif chemokine ligand 19	COL4A1	collagen, type IV, alpha 1	CD40LG	CD40 ligand	CD86	CD86 molecule	PEBP1	phosphatidylethanolamine binding protein 1
CD2	CD2 molecule	ENPP2	ectonucleotide pyrophosphatase/phosphodiesterase 2	CD274	CD274 molecule	CD160	CD160 molecule	SOCS1	suppressor of cytokine signaling 1
CD8A	CD8a molecule	HSPG2	heparan sulfate proteoglycan 2	FOXP3	forkhead box P3	CD244	CD244 molecule, natural killer cell receptor 2B4	TFRC	transferrin receptor
CD19	CD19 molecule	IGFBP7	insulin-like growth factor binding protein 7	FTH1	ferritin heavy chain 1	CTLA4	cytotoxic T-lymphocyte-associated protein 4		
CD34	CD34 molecule	KMT2C	lysine methyltransferase 2C; also known as MLL3	GATA3	GATA binding protein 3	IL7R	interleukin 7 receptor		
CD52	CD52 molecule	LAMB1	laminin, beta 1	HAMP	hepcidin antimicrobial peptide	KAT2B	lysine acetyltransferase 2B		
CD68	CD68 molecule	MFNG	MFNG O-fucosylpeptide 3-beta-N-acetylglucosaminyltransferase	HAVCR2	hepatitis A virus cellular receptor 2; also known as TIM3	KLRC1	killer cell lectin like receptor C1		
CD83	CD83 molecule	MSL3	male-specific lethal 3 homolog	HGF	hepatocyte growth factor	KLRG1	killer cell lectin like receptor G1		
CXCL8	C-X-C motif chemokine ligand 8; also known as IL8	OPN3	opsin 3	IFNG	interferon gamma	LAG3	lymphocyte-activation gene 3		
CXCL9	chemokine (C-X-C motif) ligand 9	PAK2	p21 protein (Cdc42/Rac)-activated kinase 2	IL2	interleukin 2	LILRB4	leukocyte immunoglobulin like receptor B4		
CXCL10	chemokine (C-X-C motif) ligand 10	RGS5	regulator of G-protein signaling 5	IL6	interleukin 6	PDCD1	programmed cell death 1		

DHRS9	dehydrogenase/reductase (SDR family) member 9	SELP	selectin P	IL10	interleukin 10	PTGER2	prostaglandin E receptor 2
FABP5	fatty acid binding protein 5	S1PR1	sphingosine-1-phosphate receptor 1	IL17A	interleukin 17A	TNFRSF9	TNF receptor superfamily member 9
GBP2	guanylate binding protein 2, interferon-inducible	TRPV2	transient receptor potential cation channel, subfamily V, member 2	LRRC32	leucine rich repeat containing 32; also known as GARP		
GPNMB	glycoprotein (transmembrane) nmb			RORC	RAR related orphan receptor C		
GZMB	granzyme B			TBX21	T-box 21		
HLA-DMA	major histocompatibility complex, class II, DM alpha						
HLA-DQB1	major histocompatibility complex, class II, DQ beta 1						
HLA-DRA	major histocompatibility complex, class II, DR alpha						
HLA-F	major histocompatibility complex, class I, F						
IL18BP	interleukin 18 binding protein						
IL32	interleukin 32						
IRF1	interferon regulatory factor 1						
ITM2A	integral membrane protein 2A						
LYZ	lysozyme (renal amyloidosis)						
MMP9	matrix metallopeptidase 9						
PARVG	parvin gamma						
PLA2G7	phospholipase A2 group VII						
RFX5	regulatory factor X5						
SLC1A3	solute carrier family 1 member 3						
STAT1	signal transducer and activator of transcription 1						
TAP1	transporter 1, ATP-binding cassette, sub-family B (MDR/TAP)						
TGFB1	transforming growth factor beta 1						

TK1	thymidine kinase 1
TOP2A	topoisomerase (DNA) II alpha
TYMS	thymidylate synthetase
UBD	ubiquitin D

Supplementary Table S2: AUROC analyses of non-invasive serum markers.

	HCMini			*Graft Fibrosis*			*Transcriptional signature of cTCMR*		
	AUC	*95% Confidence interval*		AUC	95% Confidence interval		*AUC*	*95% Confidence interval*	
**M65**	**0.621**	**0.510**	**0.732**	**0.629**	**0.502**	**0.757**	**0.679**	**0.530**	**0.828**
**AST**	0.611	0.494	0.728	0.558	0.433	0,683	0.633	0.468	0.798
**ALT**	0.580	0.463	0.696	0.555	0.432	0,678	**0.804**	**0.694**	**0.914**
**ALP**	**0.638**	**0.530**	**0.746**	**0.708**	**0.602**	**0.815**	0.621	0.450	0.792
**gGT**	0.571	0.460	0.682	**0.636**	**0.514**	**0.758**	0.602	0.393	0.812

Supplementary Table S3: Diagnostic fidelity of DSA for detection of subclinical graft injury.

	any DSA		any class II DSA	
	MFI > 1,000	MFI > 5,000	MFI > 1,000	MFI > 5,000
**Association of DSA with fibrosis**:				
OR (95% CI)	4.2 (1.7-10.7)	3.6 (1.9-6.9)	3.3 (1.3-8.4)	3.7 (1.4-10.2)
Presence of DSA predicted fibrosis	33%	33%	32%	36%
Absence of DSA excluded fibrosis	89%	87%	87%	87%
**Association of DSA with HCMini**:				
OR (95% CI)	0.19 (0.05-0.67)	0.20 (0.05-0.91)	0.08 (0.01-0.59)	0.1 (0.01-0.77)
Presence DSA excluded HCMini	92%	93%	97%	96%
Absence of DSA predicted HCMini	31%	30%	28%	28%

Supplementary References

1 Höfer, A. *et al.* DSA Are Associated With More Graft Injury, More Fibrosis, and Upregulation of Rejection-associated Transcripts in Subclinical Rejection. *Transplantation* **104**, 551-561, doi:10.1097/TP.0000000000003034 (2020).

2 Taubert, R. *et al.* Hepatic Infiltrates in Operational Tolerant Patients After Liver Transplantation Show Enrichment of Regulatory T Cells Before Proinflammatory Genes Are Downregulated. *American journal of transplantation : official journal of the American Society of Transplantation and the American Society of Transplant Surgeons* **16**, 1285-1293, doi:10.1111/ajt.13617 (2016).

3 Bohne, F. *et al.* Intra-graft expression of genes involved in iron homeostasis predicts the development of operational tolerance in human liver transplantation. *The Journal of clinical investigation* **122**, 368-382 (2012).

4 McKinney, E. F., Lee, J. C., Jayne, D. R., Lyons, P. A. & Smith, K. G. T-cell exhaustion, co-stimulation and clinical outcome in autoimmunity and infection. *Nature* **523**, 612-616, doi:10.1038/nature14468 (2015).

5 Bonaccorsi-Riani, E. *et al.* Molecular Characterization of Acute Cellular Rejection Occurring During Intentional Immunosuppression Withdrawal in Liver Transplantation. *American journal of transplantation : official journal of the American Society of Transplantation and the American Society of Transplant Surgeons* **16**, 484-496, doi:10.1111/ajt.13488 (2016).

6 Sis, B. *et al.* Endothelial gene expression in kidney transplants with alloantibody indicates antibody-mediated damage despite lack of C4d staining. *Am J Transplant* **9**, 2312-2323, doi:10.1111/j.1600-6143.2009.02761.x (2009).

Supplemental Figure S1

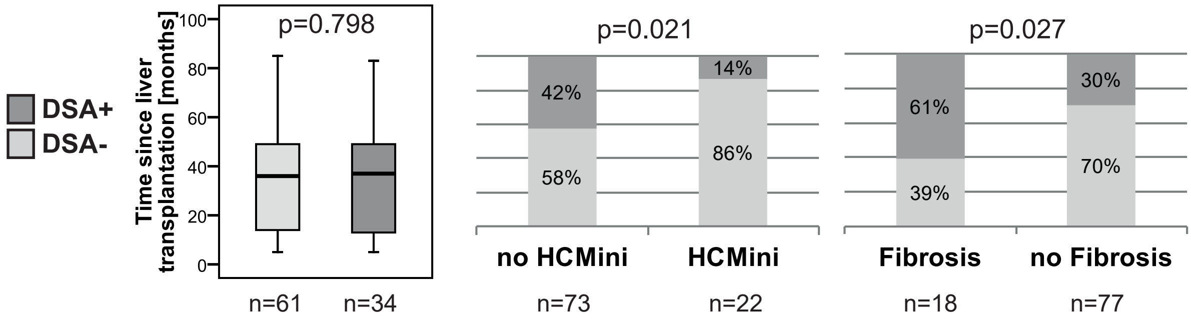

Supplement: Supplementary file 1 — Supplementary file1. [file 41598_2020_70938_MOESM1_ESM.pdf]
